# Dural reconstruction in the literature: A CiteSpace visualized bibliometric analysis

**DOI:** 10.1007/s10143-025-04124-6

**Published:** 2026-01-29

**Authors:** Erion Sulaj, Jake Barsch, John L. Kilgallon, Robert Kamil, Nitesh V. Patel, Ira M. Goldstein

**Affiliations:** 1 Department of Neurosurgery, Hackensack Meridian School of Medicine, 123 Metro Blvd, Nutley, NJ 07110 USA; 2 Department of Neurosurgery, University of Miami, Coral Gables, FL USA; 3 Department of Neurosurgery, HMH-Jersey Shore University Medical Center, Neptune, NJ USA; 4 Department of Neurosurgery, Hackensack University Medical Center, Hackensack, NJ USA

**Keywords:** Duraplasty, Bibliometrics, Cocitation, Alloplastic material, Autologous bone, Cranial reconstruction

## Abstract

Duraplasty (DP) research has shown that surgical approach to dura mater reconstruction is not standardized. The aim of this study is to explore the development of research in duraplasty from a bibliometric perspective, with a focus on literature that discusses material choice. This can elucidate trends in DP literature over time to examine and promote future research directions. Original studies and review articles on duraplasty material related research were obtained from the Scopus database from 2003 to 2024. VOSviewer software was applied to conduct both co-authorship and co-occurrence analyses. CiteSpace was used to examine occurrences of top keywords, visualize collaboration networks, and identify references and keywords with the strongest citation bursts. Top co-cited title word clusters revealed “dural substitute” and “surgical outcome” as central, recurrent keywords. “Dural substitute” emerged as a highly interconnected keyword, reflecting its importance in the literature. Temporal analysis showed that “prognostic factors” emerged consistently around 2008 and “surgical outcome” gained prominence after 2010, with keywords such as “adverse event” and “clinical outcome” spiking in later citation bursts. Material-related keywords including “biocompatible materials” (2006–2011) and “dural substitute” (2011–2017) remained consistent, with “collagen” as the most frequently mentioned non-autologous material. This bibliometric analysis highlights a literature shift from procedural considerations to surgical outcomes and material selection. Similarly, the growth of outcome-based keywords reveals increased scholarly focus on outcomes relevant to patient care. These findings emphasize the need for a standardized approach to material selection in DP. Future studies should continue to evaluate long-term outcomes and patient satisfaction to optimize dural reconstruction techniques.

## Introduction

Dural reconstruction, or duraplasty, is a surgical procedure to restore the integrity and function of the outermost layer of tissue surrounding the brain and spinal cord [1, 2]. The dura mater plays an important role in mechanical protection, cerebrospinal fluid dynamics, and the flow of blood between the brain and spinal cord [3]. Disruptions to the dura from trauma, infection, tumor growth, or surgical intervention are indications for dural reconstruction [4, 5]. Material selection for this procedure is highly relevant, as the chosen graft must effectively seal the defect, prevent leaks, and integrate with the surrounding tissue [6, 7].

The materials used for dural reconstruction include autologous tissue, synthetic grafts, xenografts, and allogeneic options, each with specific benefits and drawbacks [3, 8]. Autologous tissue grafts, such as fascia lata, offer biocompatibility and low rejection rates but may be limited by size and harvesting challenges [6, 9]. Synthetic options, such as collagen matrices and expanded polytetrafluoroethylene (ePTFE), are frequently used for their availability and ease, though they vary in durability, integration, and risk of infection [3, 10]. Further, allogeneic grafts and xenografts provide an alternative to autologous sources but can present compatibility concerns [11, 12]. Material selection should factor surgical location, patient-specific needs, and potential complications [6, 13]. Current literature shows discrepancies on how to assess this criteria [2, 3].

Bibliometric analysis is an effective tool for assessing trends and developments in dural reconstruction research, particularly regarding material selection and surgical techniques [14]. Bibliometrics utilize mathematical and statistical methods to identify citation relationships between articles, high impact factors at specific points in time, and topic clusters. CiteSpace, a bibliometric visualization tool, generates co-citation networks among authors, countries, keywords and. This enables the tracing of the evolution of core concepts within the field of duraplasty. CiteSpace’s visualization reveals patterns in keyword usage, shifts in research focus, and the contributions of different regions, providing a knowledge landscape in dural reconstruction.

This study applies bibliometric analysis to the past twenty years of duraplasty literature to uncover collaboration networks, influential publications, and emerging research gaps. Such insights can offer valuable guidance for future studies and improve outcomes in dural reconstruction.

## Methods

### Data source

Scopus was used for data retrieval, as our data analysis tool only accepts input from a single database. Scopus was chosen due to its wider coverage of academic literature than the Web of Science and its recognition as the largest abstract and citation database in the world [15]. Therefore, it likely provides broader coverage of relevant literature than other database options.

All data was retrieved November 20, 2024 from the Scopus database. The literature search parameters were set with a focus on related keywords within the title, abstract, or indexed keywords of each article. Each article was required to mention one of “duraplasty”, “dural reconstruction”, or “dura mater reconstruction” in their abstract or in their indexed keywords. This search was meant to yield a high number of studies with minimal automated exclusion. Specific materials were not included in the initial database search criteria, as many articles did not mention the material used directly in the abstract. Instead, DP material specifics were manually screened after filtering the dataset.

**Fig. 1:**
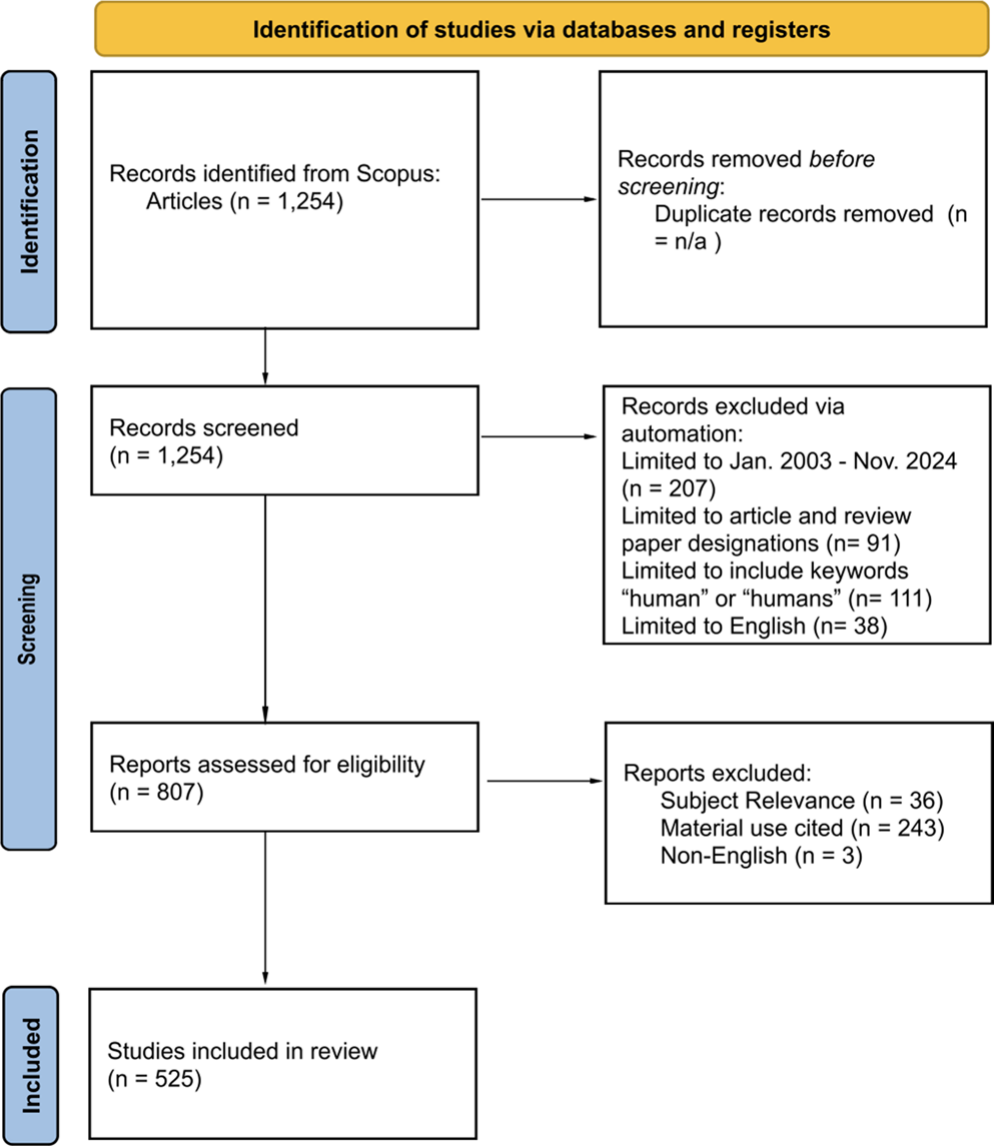
PRISMA Diagram. 1,254 articles were identified by database search. This was then reduced to 807 articles via an automated exclusion. After screening for eligibility, 525 articles remained. These articles were extracted and exported for analysisz

Figure 1 is a PRISMA diagram depicting the identification of studies for this project. Our initial search yielded 1,254 results. We then filtered to our chosen time frame of 2003–2024, which reduced the results to 1,047. We included both articles and review papers to better trace co-citations and sources, as review articles can be cited in lieu of their original study counterparts. This limited the results to 956. We then filtered for the keywords “human” or “humans” to exclude animal study results, as well as English language publications, which left 807 articles. The exact search query was as follows: TITLE-ABS-KEY (“dura mater reconstruction”) OR TITLE-ABS-KEY (“duraplasty”) OR TITLE-ABS-KEY (“dural reconstruction”) AND PUBYEAR > 2003 AND PUBYEAR < 2025 AND (LIMIT-TO (DOCTYPE, “ar”) OR LIMIT-TO (DOCTYPE, “re”)) AND (LIMIT-TO (LANGUAGE, “English”)) AND (LIMIT-TO (EXACTKEYWORD, “Human”) OR LIMIT-TO (EXACTKEYWORD, “Humans”)).

The remaining articles were then individually analyzed to ensure they met the inclusion criteria (1) human study/review, (2) the article is in english, (3) relevant to duraplasty analysis, (4) the type of material used for dural reconstruction. Many of the articles did not meet the fourth criteria, stating that a material was used but not clarifying which material at any point in their respective manuscripts. This caused significant elimination. To preserve mapping integrity, articles that did not mention an explicit type of material, but had a high field-rated impact score (> 2.00), were included in the final dataset. This ensured our citation analysis represented the full scope of the duraplasty research topic. A total of 525 articles were exported to CiteSpace for analysis.

### Data analysis

CiteSpace (6.3.R3) was used to convert the format of the included documents to a readable text file, and calculate centrality of authors, keywords, countries, and co-cited references. This information was then visualized in chronological maps to show clustering, connections, and reveal “bursts” in certain keywords and cited references, which reflect periods of increasing scholarly attention. The years per slice function was set to 1 year, to maximize temporal resolution and detect year-to-year changes in citation behavior. A K value of 25 was set for statistical analysis with the top N method for node selection, consistent with prior bibliometric studies, to balance network complexity with interpretability for statistical analysis. Different data groupings, mentioned above, were selected to highlight distinct domains. Frequency scores were restricted to 4 + for keywords and 45 + for co-cited authors to reduce noise and enhance network clarity. Clusters were created using the log-likelihood ratio (LLR) method, identifying high internal consistency and clear thematic labeling. Node size was proportional to strength of correlation for visual emphasis. Mapping metrics were set to betweenness centrality, Kleinberg’s burst detection algorithm, and the Pathfinder algorithm to reduce redundant links. All bibliometric analyses and visualizations were generated from this single dataset spanning 2003–2024, with data retrieved on November 20, 2024.

## Results

### Keyword analysis

**Fig. 2:**
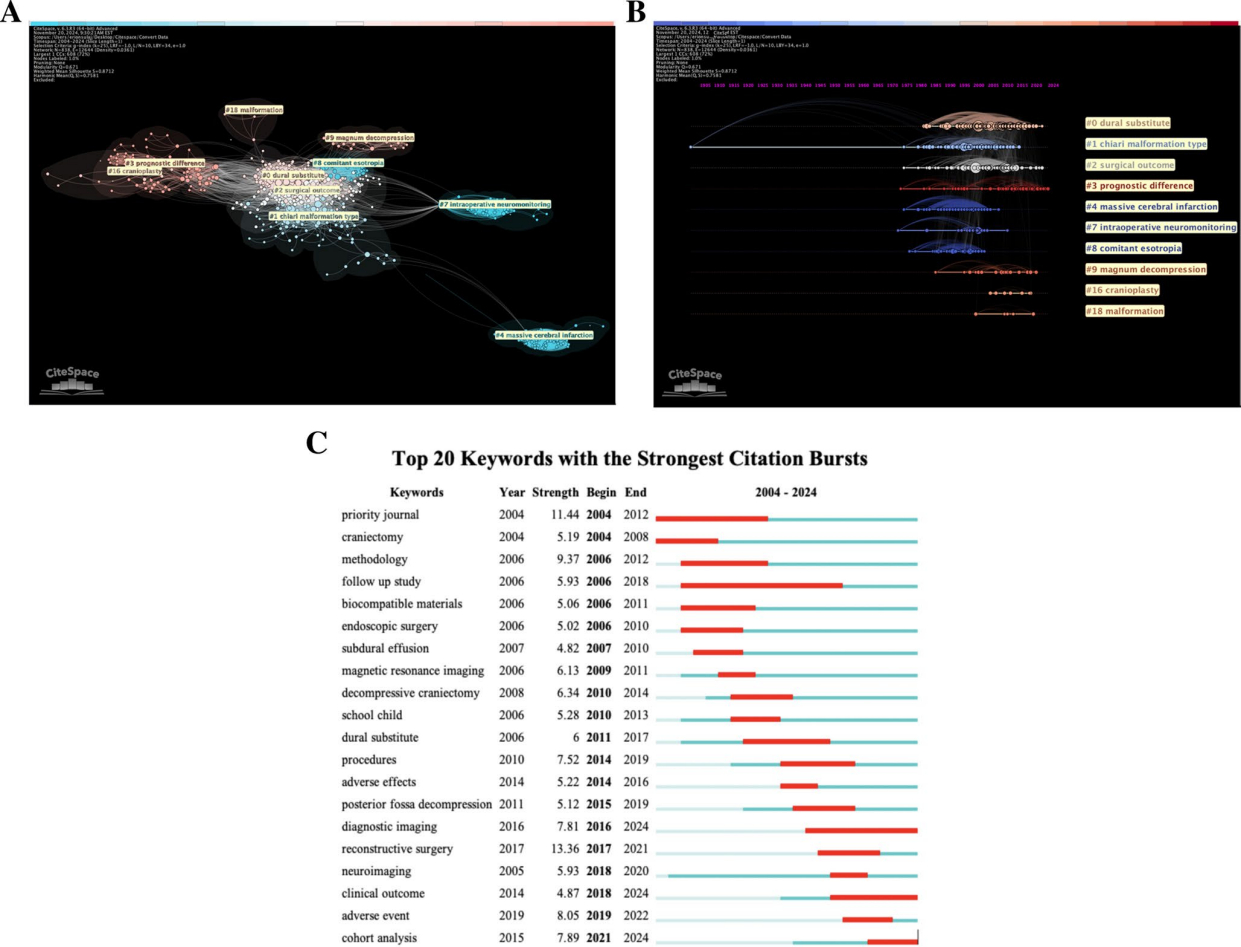
Top Keyword Characteristics. **A**. Top co-cited keyword web. There are 10 identified keyword clusters with significant modularity and silhouette scores (Q=0.671; S=0.8712). “Chiari malformation” and “surgical outcome” were highly interconnected with “dural substitute” and together they make up the top 3 most commonly occurring keywords in the dataset. **B**. Top Keyword Timeline. The top keywords are represented in a timeline map, which shows their emergence and popularity over time. Node size represents increased usage. Line tracings represent citation connections between the keywords. Node density and citation tracing is most notable for the top 3 keywords; “dural substitute,” “chiari malformation,” and“surgical outcome.” **C**. Top keyword citation bursts. Keywords with strong bursts in occurrence within the literature are documented with their burst strength and timing. Methodology-related keywords have earlier bursts, while outcomes-related keywords burst later

Top co-cited title word clusters were mapped in relation to each other (Fig. 2A). We identified 10 clusters in this cocitation reference network with significant modularity and silhouette scores indicating clusters that are reliable (Q = 0.671; S = 0.8712). Clusters with little overlap include “massive cerebral infarction,” “intraoperative neuromonitoring,” and “malformation,” suggesting that the relation of duraplasty to these clusters is specific. However, clusters such as “dural substitute,” “surgical outcome,” and “chiari malformation type 1” have considerable overlap, which reveals them as common among duraplasty articles. The strength of each cluster, depicted by the density of colored lines, shows “dural substitute” and “surgical outcome” as title words with high recurrence among articles. Cluster #0 (dural substitute) represents a central keyword that is interconnected among the other top keywords and articles. The identification of “dural substitute” as a central keyword suggests that the data extraction strategy captured literature focused on duraplasty materials, consistent with the aims of this study.

The recurrence of the top co-cited keywords and title words over time was also assessed (Fig. 2B). The size of the nodes represent the amount of citations at a specific point in time, with smaller lines linking citation links among keywords. This affords a unique perspective on how these keywords developed individually along with changes in the duraplasty. “Chiari malformation type 1” has the earliest clusters, with its greatest occurrence around 1995 before a sudden drop off. “Dural substitute” shows consistent strength from the 1980’s through the present day, highlighting the relevancy of dural substitute research as an important and evolving field. “Surgical outcome” also appears in this timeframe, but its node size begins to expand significantly in 2010. “Prognostic difference” appears around 2008 and remains consistent through the present day. This suggests a change in scholarly focus, with procedural considerations of duraplasty supplemented by increased attention to the indications and outcomes of the procedure. It is important to highlight the single co-citation dated 1900 (Fig. 2B) as a software generated artifact. This reference was not included in the original dataset, but instead, CiteSpace automatically mapped it as a historically co-cited reference associated with included articles. Due to current software constraints, such co-citation artifacts cannot be selectively removed. To the best of our knowledge, current limitations in the software do not allow for retroactive co-citation identification outside of our dataset, nor does it allow us to remove the datapoint from the figure.

Bursts were assessed to understand changes in relevance (Fig. 2C). These bursts are timeframes where a keyword was used statistically greater than average in our data set. Burst strength correlates to the relative quantity of a specific increase in publications for a given keyword. A higher score indicates that there was a more significant peak compared to other bursts. Keywords regarding surgical complications and outcomes burst later in our dataset, which is consistent with the top keyword timeline. For example, “biocompatible materials” bursts from 2006 to 2011 and “dural substitute” bursts from 2011 to 2017. However, additional later bursts including “adverse event” and “clinical outcome” in the late 2010’s indicate a growing literature emphasis on outcomes associated with duraplasty procedures. There was a strong focus on surgical strategies earlier on as “methodology” bursts from 2006 to 2012. “Reconstructive surgery” has the strongest burst among all keywords with a score of 7.47 from 2017 to 2020. Coinciding bursts of “neuroimaging” and “diagnostic imaging” suggest an uptick in focus of imaging for duraplasty, likely related to increased presurgical planning and outcome-oriented care.

Table 1 depicts the top 27 relevant keywords regarding dural reconstruction materials. “Dura mater” and “duraplasty” were the most common keywords among all articles, which was expected considering they are vital to the focus of this paper. The most common material keywords focus on autologous duraplasty with “bone graft”, “autograft”, and “autotransplantation” occurring significantly more than other materials such as “polytetrafluoroethylene” and “surgical mesh”. However, collagen was the most common nonautologous material among the articles and has more mentions than “autograft” and “autotransplantation.”

**Table 1:** Top relevant keyword frequency and centrality

Rank	Keyword	Frequency	Centrality
1	dura mater	146	0.04
2	duraplasty	97	0.03
3	computer assisted tomography	56	0.02
4	fibrin glue	22	0
5	transplantation	21	0
6	bone graft	20	0
7	dural substitute	13	0
8	antibiotic agent	11	0
9	autograft	11	0
10	collagen	11	0
11	autotransplantation	9	0
12	fascia	9	0
13	polytetrafluoroethylene	6	0
14	surgical glue	5	0
15	allograft	5	0
16	surgical mesh	5	0
17	surgical flaps	5	0
18	dural graft	4	0
19	dural reconstruction	4	0
20	expansile duraplasty	4	0

The top 20 keywords occurring in the dataset are listed with their exact frequency and centrality scores

### Citation and publication analysis

**Fig. 3:**
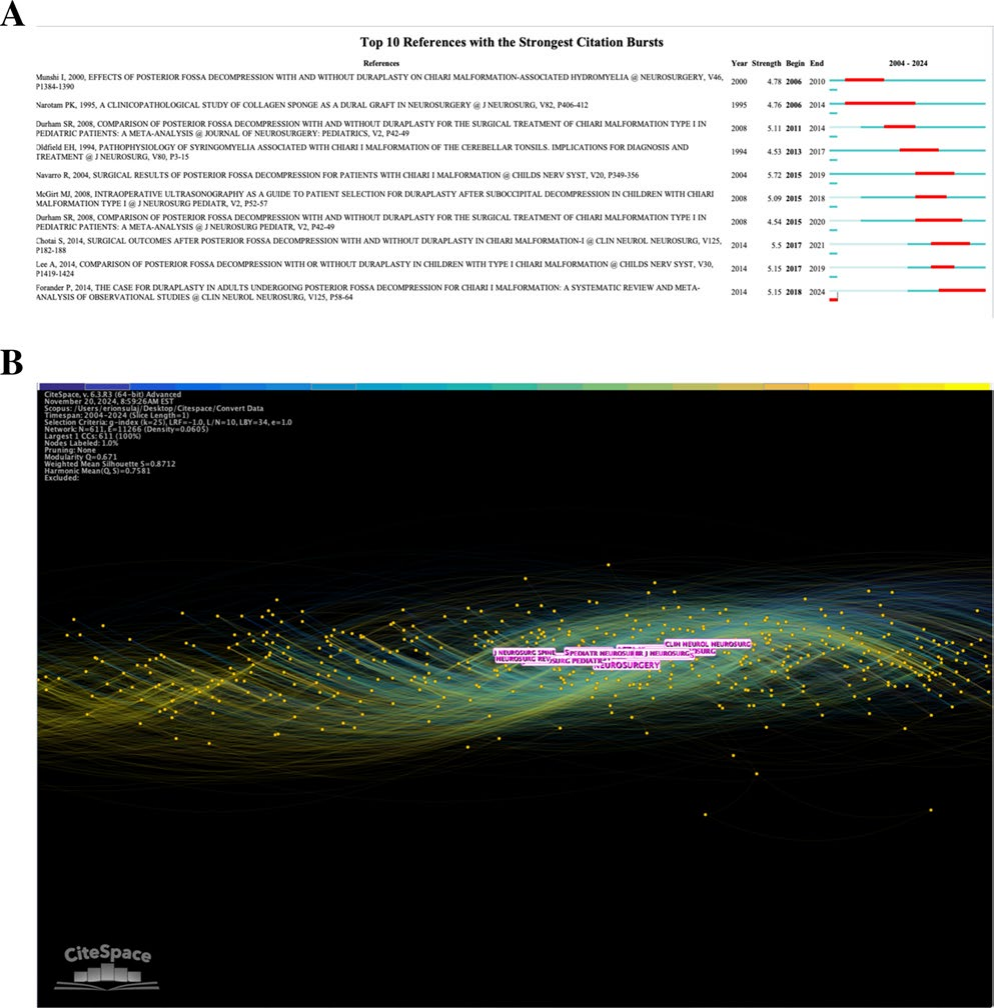
Top citation and publication characteristics.** A**. Top 10 references with strongest citation bursts. Specific references with strong citation bursts are depicted with their burst strength and timing. Notably, most references concern Chiari malformation, which may represent a skew in citation mapping. **B**. Chronological journal publishing web. Journals with articles concerning DP materials are visualized in a spatial map. The single cluster implies general centrality among all journals contributing to the dataset

To better understand these citation relationships, articles with the strongest citation bursts in the last 20 years were assessed (Fig. 3A). Chiari I malformation articles were the most common, accounting for nine of the 10 articles with the largest citation bursts, consistent with the results in Table 2. It is notable that the articles with earlier bursts focus on surgical strategies, while the two articles with the most recent bursts assess surgical complications, again in line with the results from Fig. 2A, B and C.

**Table 2:** Most co-cited articles

Rank	Title	First Author	Frequency	Centrality	Year	Source journal
1	Effects of posterior fossa decompression with and without duraplasty on Chiari malformation-associated hydromyelia	Munshi I.	24	0.09	2000	Neurosurgery
2	Comparison of posterior fossa decompression with and without duraplasty for the surgical treatment of Chiari malformation Type I in pediatric patients: a meta-analysis	Durham SR.	23	0.14	2008	Journal of Neurosurgery
3	Surgical results of posterior fossa decompression for patients with Chiari I malformation	Navarro R.	21	0.05	2004	Child’s Nervous System
4	Chiari I malformation redefined: clinical and radiographic findings for 364 symptomatic patients	Milhorat TH.	20	0.09	1999	Neurosurgery
5	Surgical outcomes after posterior fossa decompression with and without duraplasty in Chiari malformation-I	Chotal S.	18	0.11	2014	Clinical Neurology and Neurosurgery
6	Suboccipital decompression for Chiari I malformation: outcome comparison of duraplasty with expanded polytetrafluoroethylene dural substitute versus pericranial autograft	Attenello FJ.	16	0.09	2009	Child’s Nervous System
7	Institutional experience with 500 cases of surgically treated pediatric Chiari malformation Type I	Tubbs RS.	16	0.05	2011	Journal of Neurosurgery
8	Comparison of dural grafts in Chiari decompression surgery: Review of the literature	Abla AA.	14	0.08	2010	Journal of Craniovertebral Junction and Spine
9	Duraplasty or not? An evidence-based review of the pediatric Chiari I malformation	Hankinson T.	14	0.04	2011	Child’s Nervous System
10	The case for duraplasty in adults undergoing posterior fossa decompression for Chiari I malformation: a systematic review and meta-analysis of observational studies	Forander P.	14	0.06	2014	Clinical Neurology and Neurosurgery

The 10 articles with the most co-citations in the dataset with their exact frequency and centrality scores

The article with the most recent burst was published in 2014 but did not burst until 2018. This is an indicator that while the focus of scholarly discourse may be evolving, the methodology used to understand duraplasty is still relevant. Valid early publications provide a sound basis for conclusions to be drawn upon for future directions in duraplasty. The ten most co-cited articles were determined in Table 2. Again, nine of ten articles focus on Chiari I malformation, which is significant considering the wide range of applications for duraplasty. However, no article in the table was published more recently than 2014, so the more recent developments in duraplasty may not be fully recognized. Likewise, an increase in meta-analyses may hinder the citation counts for newer articles.

Figure 3B depicts publications by journal over time. The central cluster of titles indicates a lack of a predominant journal in publishing duraplasty articles. The circular nature of the visualization means that each journal is responsible for several contributions to our data set. Table 3 recorded the publication details of the top journals and their citation frequencies. *Neurosurgery* and the *Journal of Neurosurgery* accounted for the greatest number of publications, with two pediatric journals contributing to the top six. *Neurosurgery* had an incredibly high centrality score of 0.56, revealing a strong link among its citations. This can be attributed to both a high number of co-citations among articles published by the journal and a common focus in the articles that the journal publishes. Such a high centrality score is a strong outlier, with most of the top ten journals scoring below 0.10.

**Table 3:** Journal frequency and centrality

Rank	Title	Frequency	Centrality
1	Neurosurgery	219	0.56
2	Journal of Neurosurgery	194	0.18
3	World Neurosurgery	133	0.09
4	The Journal of Neurosurgery: Pediatrics	114	0.11
5	Childs Nervous System	110	0.06
6	Acta Neurochirurgica (Wien)	93	0.06
7	Clinical Neurology and Neurosurgery	87	0.05
8	Neurosurgical Focus	83	0.07
9	Neurosurgical Review	81	0.08
10	Surgical Neurology	79	0.06

The 10 journals with the most co-citations in the dataset are depicted with their exact frequency and centrality scores

### Contribution analysis

**Fig. 4:**
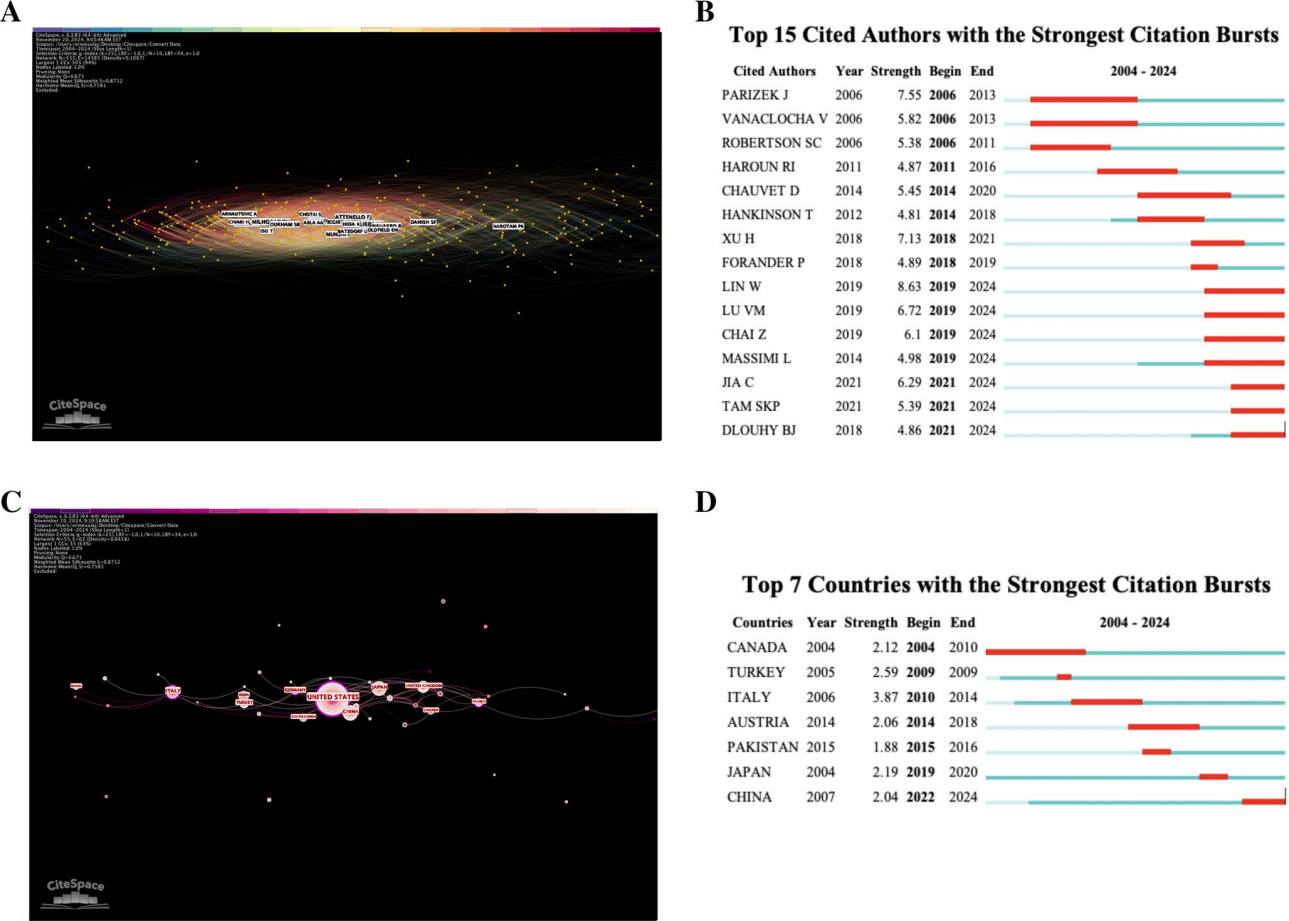
Authorship characteristics.** A**. Author chronological co-citation web. Individual authors with significant recurrence in the dataset are mapped. Node connections depict inter connectedness between author citations among articles within the dataset. **B**. Top 15 authors with strongest citation bursts. Individual authors with strong bursts in citation frequency are deported with their burst strength and timing. **C**. Publications by country. Citations by country of origin are depicted in this visualization. The United States has both the largest node and is the most center within the figure, implying a leading number of publications and central occurrence within the dataset. **D**. Top 7 countries with strongest citation bursts. Countries with citation bursts are depicted with their burst strength and timing

The contributions of different authors from 2003 to 2024 was determined (Fig. 4A), with node connections equating to the amount of co-citations received by different authors. Citation bursts by individual authors can be used to assess impact over the last decade (Fig. 4B). Authors including Parizek, J., Vanaclocha V., and Robertson SC. had early bursts with strengths > 4.5, a trend that remained consistent with more recent bursts. The author with the strongest citation burst was Lin, W., with a score of 8.63 from 2019 to 2024 which is significant. A consistency of strong bursts throughout the years may be explained by similar research focuses, or a steady amount of collaboration occurring between authors.

Further, Table 4 lists the ten authors with the greatest number of co-citations in our data set, where it is notable that none of the top authors appear in the citation burst analysis. Tubbs, RS. tops the list of citations with a relatively high centrality score of 0.16 in comparison with the other top authors. This indicates a greater impact of his contributions to the field of duraplasty. His article *Surgical experience in 130 pediatric patients with Chiari I malformations* appears in Table 2 with 17 co-citations. However, the lack of an identifiable “foundational” article likely prevents this conclusion.

**Table 4:** Individual author frequency and centrality

Rank	Author	Frequency	Centrality
1	Tubbs RS.	86	0.14
2	Klekamp J.	73	0.05
3	Milhorat TH.	56	0.05
4	Durham SR.	50	0.07
5	Attenello FJ.	47	0.06
6	Chotai S.	42	0.06
7	Mcgirt MJ.	38	0.02
8	Munshi I.	37	0.01
9	Arnautovic A.	35	0.02
10	Navarro R.	35	0.02

The 10 authors with the most co-citations in the dataset are depicted with their exact frequency and centrality scores

Duraplasty material literature was also mapped by country of authorship (Fig. 4C). Since 2003, the United States has contributed the greatest amount of papers with over 195 individual publications. China (*n* = 51), Japan (*n* = 50), Italy (*n* = 45), and Turkey (*n* = 33) make the subsequent greatest contributions (Table 5). The United States overall centrality score is 0.52, revealing a strong level of direct collaboration within the country. Similarly, Italy and France have centrality scores > 0.15, indicating significant intra-national collaboration. The low centrality scores of China, Turkey, India, and France (< 0.1) suggests non-significant collaboration with other nations. Figure 4D examined citation bursts by country of authorship. Italy had the strongest burst between 2010 and 2014, indicating a geographical peak in Duraplasty research. However, each of the top seven bursts have strengths of < 4.0 implying a lack of a true peak in duraplasty interest in a country dependent manner.

**Table 5:** Country frequency and centrality

Ranking	Country	Frequency	Centrality
1	United States	195	0.52
2	China	51	0
3	Japan	50	0.06
4	Italy	45	0.22
5	Turkey	33	0.01
6	Germany	27	0.12
7	United Kingdom	22	0
8	India	19	0
9	France	14	0.17
10	Canada	13	0.05

The 10 countries with the most co-citations in the dataset are depicted with their exact frequency and centrality scores

## Discussion

Bibliometric analysis can show important research trends through its ability to perform co-occurring author keyword analyses and keywords bursts, providing insight to the most relevant keywords identified by researchers at various time points and reflecting the key areas of focus during those periods [16]. For example, a bibliometric analysis of atlantoaxial spine surgery articles examined the use and evolution of various internal fixation techniques and highlighted the growth and impact of new procedures while outlining those that have been largely abandoned. The wide scope of this analysis made a strong impact on future studies, supported by its field-weighted impact score of 9.07, by offering a point of reference for future research to build from [17].

The consistency and centrality of “dural substitutes” reflects its pivotal role in both historical and current surgical discourse. While “surgical outcome” followed a similar pattern, the prominence of materials-focused keywords suggests that optimal graft material remains a core question in the existing body of literature. This finding aligns with prior formal meta-analyses and reviews that emphasize the ongoing debate of autologous versus synthetic materials in terms of optimal outcomes [18–20].

The observed shift in keyword bursts from surgical strategies in the early 2000 s to surgical methodology a decade later to surgical outcomes in recent years suggests a maturation in research interests from technique development toward evaluating procedural effectiveness and relevant outcomes. This progression aligns with broader scholarly trends in neurosurgery emphasizing both innovation and subsequent refinement [21]. However, even with a change of focus, the emphasis on material related keywords remained consistent throughout the observed period.

In terms of material keyword prominence (Table 1), their distribution likely reflects the historical role of autologous tissue as the reference standard for DP. Autologous grafts are frequently cited in methodological contexts, which may explain their persistence in the literature [7, 10]. However, the frequency of collagen materials may indicate growing scholarly interest in such readily available substitutes. These patterns imply evolving research emphasis toward alternatives to autologous grafts.

Data including author, country, and journal information served as a valuable resource to analyze this progression of DP research. High impact studies, such as those by Attenello et al. on synthetic versus autologous grafts and by Durham and Munshi on posterior decompression outcomes, demonstrate that both material selection and surgical context are integral to surgical decision making [18, 22, 23]. These studies offer methodological bases for current research endeavors, and their prominence may indicate that foundational concepts to DP remain relevant. Further, geographic and journal trends provide additional insight into the field, as the United States dominance in publication volume and citation centrality may reflect a disproportionate influence on existing global DP scholarship. However, the high centrality in smaller nations such as Italy imply that targeted, high quality publications can still have a strong impact. In general, this findings aligns with a prior report that roughly 48.8% of neurosurgery literature came from the United Stated from 2011 to 2020 [24]. Similarly, the concentration of journals with a focus on pediatric neurosurgery suggests the pediatric population as a potential driver of DP innovation.

Finally, the unique centrality of the data on pediatric literature, namely Chiari I malformation, must be recognized as a potential bias in the data. Chiari is one of the most common procedures in dural reconstruction, as it has a fundamental focus on the dural/cerebrospinal fluid interface [25]. Its prominent literature focus could be due to volume, or a more consistent followup for pediatric patients, but is most likely due to the relative standard indications for DP in a Chiari decompression [26].

In contrast, DP is commonly performed in other contexts, including skull base tumor, trauma, and cerebrospinal fluid leak, yet these applications appear comparatively underrepresented. Such discrepancy may reflect greater heterogeneity in approach, and technique, as well as a research focus that prioritizes other outcomes over dural repair itself. However, these cases are often within larger operative paradigms with DP as a technical adjunct rather than an independent variable of study, limiting dedicated analysis [4]. Overall, dural material choice assumes greater clinical, technical, and investigative importance in Chiari I surgery as compared to other procedures [19, 20]. It was for this reason that articles without a specific material cited, but a high impact score, were included in the study. This was to increase the breadth of our dataset and open bibliometric analysis to popular DP literature that includes general material choice considerations to help prevent literature bias in our analysis.

Overall, these patterns reveal DP research has entered a phase where outcome optimization, particularly regarding material choice, is at the forefront of scholarly focus. Future surgeons would benefit from a clear, standardized approach to material choice, which is achievable through an expanded, multinational research approach. This could diversify previous conclusions and accelerate consensus towards best practices.

### Limitations

The limitations of this analysis stem from its scope. Our study exclusively used the Scopus database, which is highly comprehensive, but it does not include articles in other databases such as World of Science and PubMed. However, Scopus is considered the largest and most comprehensive database in the world [15, 27]. This is due to software limitations, as CiteSpace software allows for single database analysis. The data was focused exclusively on human research and english (or english-translated) studies, which should be considered when scoping early dural research in animal subjects.

In addition, relevant articles might have been missed by our search query due to a lack of specified keywords in their abstracts. This was safeguarded by including high field-weighted citation impact studies. Bibliometric analyses can also run into issues of citation lag, as research can take several months to years before publication, leaving more recent studies underrepresented in the dataset. However, our procedure falls within accepted standards and these limitations are common to bibliometric studies.

## Conclusion

This bibliometric analysis highlights key trends in duraplasty research focus, particularly a shift from procedural considerations to a focus on surgical outcomes and material selection. Autologous materials remain prominent, but non-autologous options such as collagen are increasingly discussed, reflecting evolving data for clinical practice. The recurrence of “dural substitute” as a central keyword underscores its importance in the field, while the growing literature emphasis on outcomes including “adverse event” and “clinical outcome” suggests greater interest in a patient-centered approach. These findings provide valuable insight for guiding future research in duraplasty, emphasizing a need for clear material selection criteria and further exploration of non-autologous options. Future studies should validate the identified trends with empirical studies, evaluate long-term outcomes, and optimize dural reconstruction techniques.

## Data Availability

Not applicable.
